# Rehabilitation professionals at the COVID-19 frontline: a testimony-informed narrative review of health-worker mortality, vaccine hesitancy, physiotherapy and telerehabilitation

**DOI:** 10.3389/fresc.2026.1921614

**Published:** 2026-10-21

**Authors:** Ibrahim Npochinto Moumeni

**Affiliations:** 1 Department of Physiotherapy and Physical Medicine, Faculty of Medicine and Pharmaceutical Sciences, University of Dschang, Dschang, Cameroon; 2 Physical Medicine and Osteopathy Department, Bafoussam Regional Hospital, Bafoussam, Cameroon; 3 Department of Geriatrics and Gerontology, Sorbonne Université/Pitié-Salpêtrière Hospital, Paris, France; 4 Société Africaine Francophone de Neuro-Rééducation (SAFNeR), Parakou, Benin

**Keywords:** burnout, COVID-19, health workers, narrative review, physiotherapy, post-COVID-19 condition, telerehabilitation, vaccine hesitancy

## Abstract

**Background:**

COVID-19 placed an unprecedented burden on health workers and disrupted rehabilitation services. Existing reviews have examined health-worker infection and mortality, vaccine hesitancy and telerehabilitation separately; few have considered these dimensions together from the perspective of rehabilitation professionals.

**Methods:**

Narrative review following SANRA recommendations. PubMed, Scopus and Google Scholar were searched (initial search January 2019–December 2022; updated during revision to December 2025 to capture later publications reporting 2020–2021 data). Sources were selected purposively, prioritising systematic reviews, large multicentre studies and official agency reports. First-person clinical observations from the author's postgraduate training in two French university hospitals are presented separately, in labelled boxes, and are not used as evidence for quantitative claims.

**Results:**

WHO estimated 80,000–180,000 health and care worker deaths from COVID-19 between January 2020 and May 2021 (central estimate 115,500), versus 6,643 reported through surveillance. Front-line workers had a 3.4-fold higher adjusted risk of a positive test than the general community. Vaccine hesitancy among health workers was 37% in a 91-country survey and 22.5% in a review of 76,471 workers; a scoping review of nurses reported 20.7% refusal. In a French tertiary centre, 73.1% were in favour, 23.1% hesitant and 3.9% opposed. Physiotherapy guidance addressed acute respiratory care, early mobilisation and post-COVID-19 rehabilitation. Telerehabilitation achieved outcomes similar to centre-based pulmonary rehabilitation, but accounted for only 6% of outpatient episodes in a large US database. Four heuristic constructs are proposed: Frontline Abandonment Syndrome (FAS), Vaccination Refusal Cascade (VRC), Telerehabilitation Emergency Pivot (TEP) and Post-COVID Functional Decoupling (PCFD). Each is positioned against existing terminology and presented as a testable hypothesis rather than a validated construct.

**Conclusions:**

COVID-19 exposed gaps in health-worker protection, communication around vaccination and the integration of rehabilitation into pandemic preparedness. Telerehabilitation deserves a defined place in hybrid care models, and the proposed constructs require formal concept analysis and empirical testing.

## Key points

WHO estimated 80,000–180,000 deaths among health and care workers between January 2020 and May 2021 (central estimate 115,500), whereas only 6,643 had been reported through surveillance.

In France, at the start of the vaccination campaign, 73.1% of surveyed healthcare workers favoured COVID-19 vaccination, 23.1% were hesitant and 3.9% were opposed, with a marked gradient by occupation.

Telerehabilitation achieved outcomes similar to centre-based care in several domains, yet its real-world uptake in outpatient physiotherapy remained modest (6% of episodes in a large US database).

Four heuristic constructs (FAS, VRC, TEP, PCFD) are proposed as hypotheses, explicitly positioned against burnout, moral injury, social contagion, digital transition and post-COVID-19 condition, with suggestions for how each could be tested.

The review integrates, in clearly labelled boxes, a rehabilitation practitioner's observations from two French university hospitals during 2020–2021.

## 1 Introduction

On 11 March 2020, the World Health Organization characterised COVID-19 as a pandemic, by which time more than 118,000 cases had been reported in 114 countries (1). The following two years brought hospital saturation, severe shortages of protective equipment, and rapid reorganisation of care, including rehabilitation.

Several reviews have documented specific aspects of this period: infection and mortality among health workers (2, 3), their psychological burden (4), vaccine hesitancy (5, 6) and the effectiveness of telerehabilitation (7, 8), as well as the rehabilitation needs of patients with COVID-19 (9). These reviews address each dimension separately and rarely adopt the perspective of rehabilitation professionals, who worked both in acute units and in the community.

This review aims to synthesise, for a rehabilitation readership, four interrelated dimensions:

1. health-worker mortality and withdrawal from front-line duties;
2. vaccine hesitancy among health workers;
3. the role of physiotherapy in COVID-19 care;
4. the shift to telerehabilitation, with a note on the Delta-variant period of 2021.

A secondary aim is to propose four heuristic constructs arising from this synthesis and from clinical experience, and to position them against existing concepts.

### 1.1 Author positionality

During the first pandemic waves, the author was completing postgraduate clinical training at the Henri Mondor University Hospital (Créteil) and the Raymond-Poincaré University Hospital (Garches) as part of several Master's programmes. He also provided home-based physiotherapy under the exceptional arrangements put in place by French health authorities during the first lockdown. This dual position offers a practitioner's view of events, but it is a single-observer perspective from a high-resource setting. For this reason, the observations are presented separately from the literature (Section 2.4) and are treated as illustrative rather than evidential (10).

## 2 Methods

### 2.1 Design

This is a narrative review (11), reported in line with the SANRA quality criteria for narrative reviews (12). It is not a systematic or scoping review, and it does not aim to be exhaustive.

### 2.2 Search strategy

PubMed, Scopus and Google Scholar were searched with the following terms, alone or combined with “COVID-19″ or “SARS-CoV-2″: “healthcare workers” AND (mortality OR deaths); “healthcare workers” AND (burnout OR “moral injury” OR absenteeism); “vaccine hesitancy” AND “healthcare workers”; physiotherapy OR “physical therapy”; telerehabilitation; “post-COVID-19 condition” OR “long COVID”; and “Delta variant”.

The initial search covered January 2019 to December 2022. During revision, it was updated to December 2025 to capture studies published later that reported data collected during 2020–2021. This update explains why some references are dated 2023–2024. Reference lists of key reviews were also screened.

### 2.3 Eligibility and selection

**Inclusion criteria:**

- peer-reviewed studies or official agency reports (WHO, EMA, national authorities) providing data or recommendations on the four themes;
- data collected during 2020–2021;
- publication in English or French.

**Exclusion criteria:** non-peer-reviewed preprints, and opinion pieces without data. Conceptual and ethical papers were retained only where needed to define terms. One investigative-journalism source was retained for US health-worker deaths, because no official US count exists; it is identified as such.

**Selection:** sources were selected purposively. Priority was given to systematic reviews and meta-analyses, large multicentre or national datasets, official estimates and French studies. Because selection was purposive rather than systematic, no record-screening flow diagram is reported. Instead, Table 1 lists the number and type of sources used for each theme.

**Table 1:** Sources used by theme (purposive selection; number and type).

Theme	n	Types of sources
Health-worker mortality and infection risk	6	WHO working paper and news release; 2 systematic reviews/meta-analyses; 1 prospective cohort; 1 investigative-journalism database (US)
Psychological burden, withdrawal and related concepts	8	1 meta-analysis; 2 cross-sectional surveys; 1 review of burnout; 3 conceptual papers (moral injury, compassion fatigue); 1 ethics paper
Vaccine hesitancy	13	1 definition paper; 1 multinational survey; 2 reviews; 3 French surveys; 3 regulatory/legal documents; 1 ethics paper; 2 behavioural/modelling papers
Physiotherapy and post-COVID-19 condition	7	2 clinical practice recommendations/consensus; 1 rapid living systematic review; 1 WHO survey; 2 definition/review papers; 1 PICS consensus report
Telerehabilitation	8	1 rapid overview; 1 Cochrane review; 4 observational studies; 2 commentaries on telehealth transition
Delta variant	2	1 cohort study; 1 research letter
Methodology and context	5	2 narrative-review guidance papers; 1 autoethnography overview; 1 concept-analysis textbook; 1 WHO statement
Total	49	

**Verification:** during revision, every quantitative statement was checked against the abstract or full text of the source cited.

### 2.4 Synthesis and use of clinical observations

The evidence was synthesised thematically. The author's first-person observations are presented only in shaded boxes labelled “Clinical observation”. They are recollections of the author's own practice, contain no identifiable patient information, and are not used to support any quantitative claim. The four constructs in Section 8 were derived inductively from the synthesis and these observations. They have not undergone formal concept analysis (13); this is acknowledged as a limitation.

## 3 Health-worker mortality and withdrawal from front-line duties

### 3.1 The scale of health-worker deaths

WHO estimated that between January 2020 and May 2021, 80,000 to 180,000 health and care workers died from COVID-19, with a central estimate of 115,500 (14, 15). Over the same period, only 6,643 health-worker deaths had been reported through surveillance (14), less than 6% of the central estimate.

Early data showed a marked occupational pattern. A global systematic review of data up to 8 May 2020 identified 152,888 infections and 1,413 deaths among health workers (2).

- Infections occurred mainly in women (71.6%) and nurses (38.6%).
- Deaths occurred mainly in men (70.8%) and physicians (51.4%).

A meta-analysis found that health workers made up about 10% of COVID-19 cases, with lower severity and mortality than other patients (3). In the United Kingdom and the United States, front-line workers had an adjusted 3.4-fold higher risk of a positive test than the general community, which was further increased by inadequate or reused protective equipment (16). No official count exists for the United States. An investigative project documented more than 3,600 health-worker deaths during the first year, disproportionately among lower-paid staff, including nurses and support workers (17). These estimates are summarised in Table 2 and Figure 1.

**Table 2:** Health-worker infection risk and mortality: key data (corrected attributions).

Source	Period	Estimate	Notes
WHO surveillance (14)	Jan 2020–May 2021	6,643 deaths	Acknowledged by WHO as a marked under-report
WHO analytical estimate (14)	Jan 2020–May 2021	80,000–180,000 deaths (central 115,500)	Based on 135 million health and care workers
Global systematic review (2)	To 8 May 2020	152,888 infections; 1,413 deaths	Infections: women 71.6%, nurses 38.6%; deaths: men 70.8%, physicians 51.4%
Meta-analysis, 11 studies (3)	To 23 Apr 2020	Health workers ≈10% of cases	Severity and mortality lower than in all patients
Prospective cohort, UK/USA (16)	Mar–Apr 2020	aHR 3.40 for positive test vs. general community	Higher risk with inadequate or reused PPE
Lost on the Frontline (journalistic database) (17)	First year, USA	> 3,600 deaths	No official US count; lower-paid staff, including nurses and support workers, over-represented

aHR, adjusted hazard ratio; PPE, personal protective equipment.

**Figure 1:**
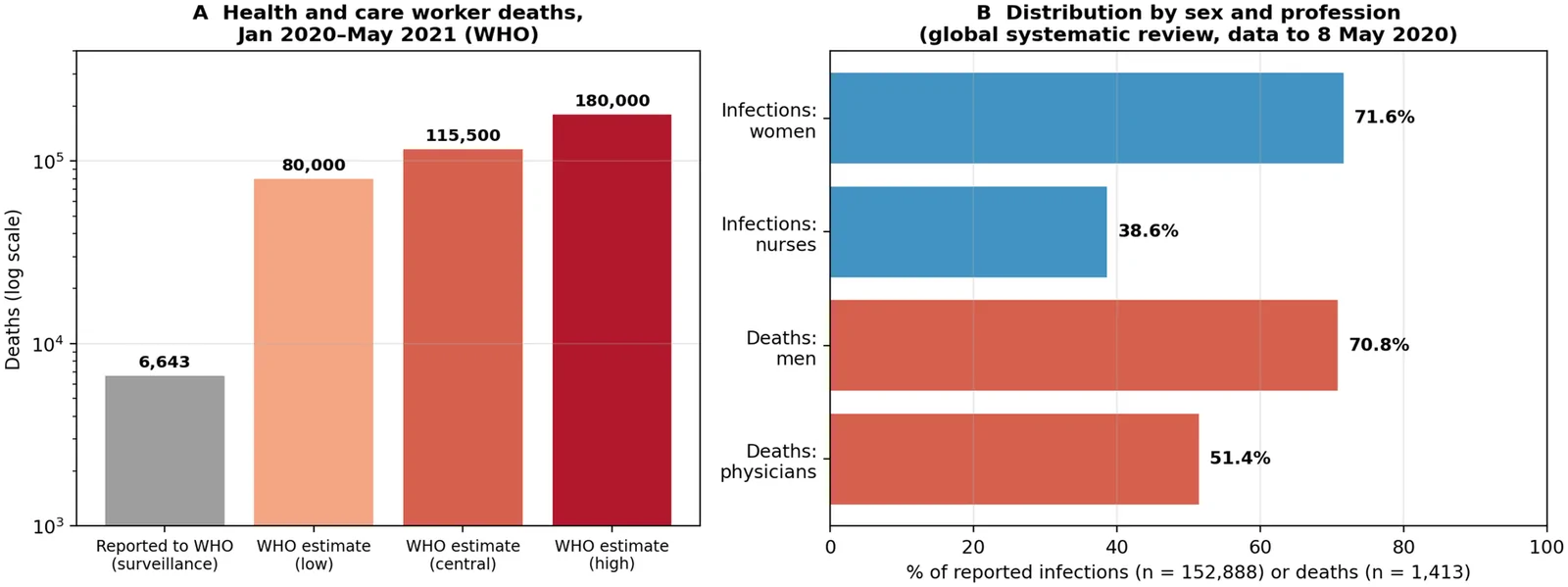
Health-nnworker deaths from COVID-19. **(A)** Deaths reported to WHO through surveillance compared with WHO analytical estimates, January 2020–May 2021 (logarithmic scale) (14). **(B)** Distribution of infections and deaths by sex and profession in a global systematic review of data to 8 May 2020; percentages refer to cases with available data (2).

### 3.2 Withdrawal from front-line duties: frontline abandonment syndrome

The psychological burden on health workers is well documented. In a meta-analysis, the pooled prevalence among health workers was 23.2% for anxiety, 22.8% for depression and 38.9% for insomnia (4). Similar distress was reported in Wuhan (18) and among French intensive-care clinicians (19). These burdens are usually conceptualised through several established constructs:

- burnout (20);
- moral injury, which arises when clinicians act, or are prevented from acting, against their moral values (21, 22);
- compassion fatigue (23).

Ethical analyses of pandemics have long discussed the limits of the duty to care when protection is inadequate (24).

Clinical observation (author). During the first wave, the author witnessed the absence of some staff members from front-line units at the time of highest demand. This occurred across professional categories, in a context of shortages of protective equipment, rapidly changing protocols and fear of contaminating relatives. These observations are those of a single trainee in two hospitals. They do not allow any estimate of the frequency of this phenomenon.

The term Frontline Abandonment Syndrome (FAS) is proposed to describe the withdrawal of health workers from high-risk front-line duties, whether temporary or prolonged, formal or informal, and its consequences for the continuity of care. It is not intended as a moral judgement. Section 8 explains how FAS differs from burnout and moral injury.

## 4 Vaccine hesitancy among health workers

### 4.1 International data

Vaccine hesitancy is defined as a delay in acceptance or refusal of vaccination despite the availability of vaccination services (25). Estimates among health workers vary with the period, the population and the definition used.

- A cross-sectional survey of 4,630 health workers from 91 countries (February–April 2021) reported an overall hesitancy rate of 37%. It was strongly associated with the Human Development Index, and physicians and pharmacists were more willing to be vaccinated (26).
- A review of studies including 76,471 health workers estimated that 22.5% were hesitant (5).
- A scoping review of 51 studies (41,098 nurses, 36 countries) found a pooled refusal prevalence of 20.7%, higher in 2020 than in 2021 (6).
- The main reasons were concerns about safety, side effects and efficacy, misinformation, and mistrust of authorities or pharmaceutical companies (6).

### 4.2 The French context

In a survey of 1,965 health workers at a French tertiary centre (February 2021), 73.1% were in favour of vaccination, 23.1% were hesitant and 3.9% were opposed (27). There was a gradient by occupation: fewer than 60% of auxiliary nurses and technicians intended to be vaccinated, compared with 60%–79% of nurses and support staff and over 80% of medical staff. The controversy over the tolerability of the AstraZeneca vaccine was independently associated with lower intention (27).

Other French surveys found similar levels of hesitancy:

- 28.4% hesitancy or reluctance among 2,678 health workers in France, French-speaking Belgium and Quebec in late 2020 (28);
- 53.2% crude willingness in a multicentre survey of 4,349 health workers, which increased over time (29).

Several events marked this period. The first COVID-19 vaccine was recommended for EU authorisation on 21 December 2020 (30). In mid-March 2021, several countries, including France, temporarily paused the AstraZeneca vaccine, until the EMA concluded on 18 March 2021 that its benefits outweighed its risks (31). The law of 5 August 2021 then made COVID-19 vaccination mandatory for health workers (32), a measure that raised ethical debate (33). These data are summarised in Figure 2.

**Figure 2:**
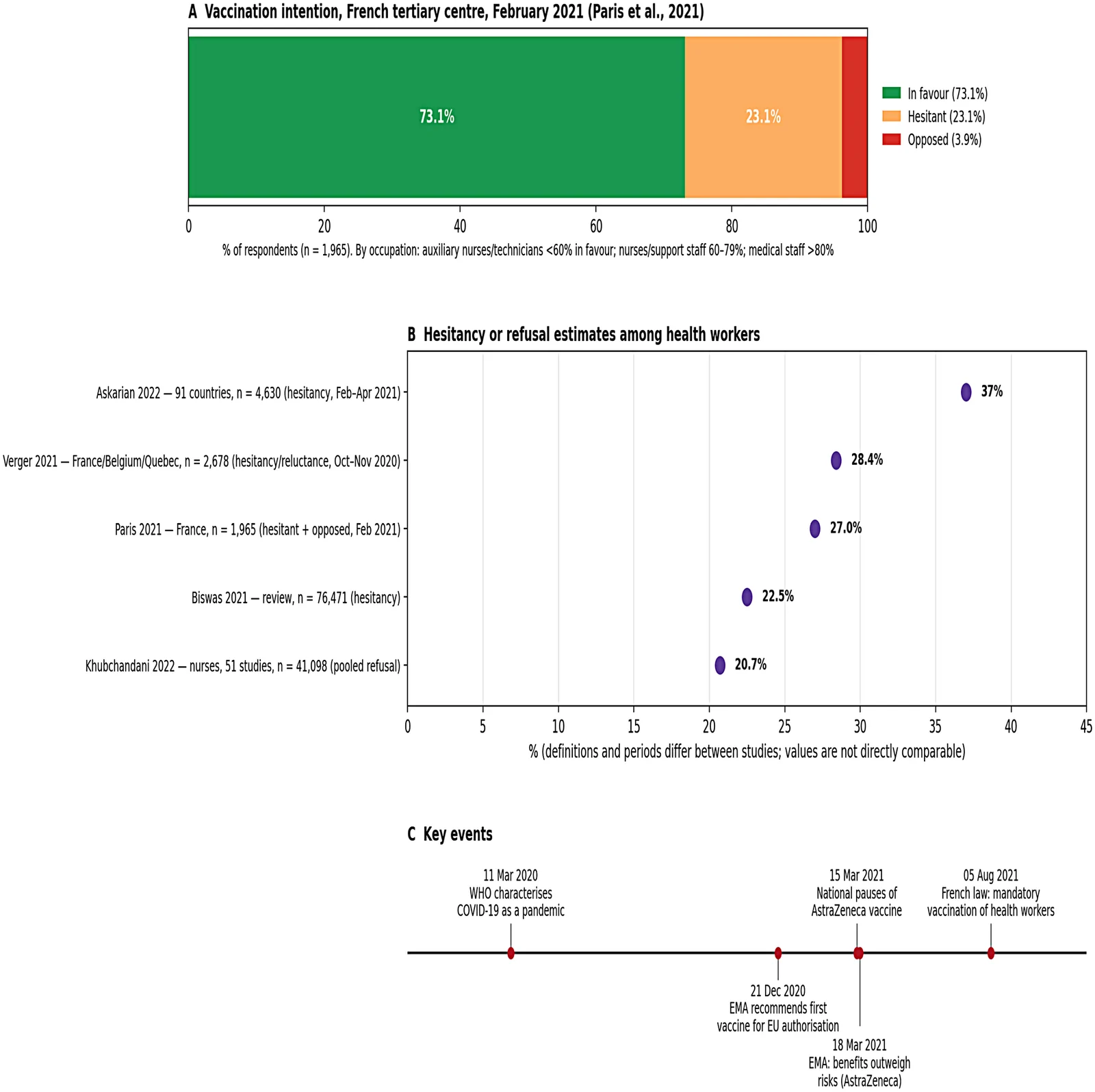
COVID-19 vaccine hesitancy among health workers. **(A)** Vaccination intention at a French tertiary centre, February 2021 (*n* = 1,965) (27). **(B)** Hesitancy or refusal estimates from the studies cited; the definitions differ between studies, so the values are not directly comparable (5, 6, 26–28). **(C)** Timeline of key events (1, 30–32).

The term Vaccination Refusal Cascade (VRC) is proposed for the hypothesised process by which a salient institutional or media event triggers successive refusal decisions within professional networks, extending hesitancy beyond people who were previously sceptical. VRC builds on models of opinion clustering and social influence (34, 35). The association between the AstraZeneca controversy and intention in French data (27) is consistent with, but does not demonstrate, such a process.

## 5 Physiotherapy during the COVID-19 pandemic

### 5.1 Acute and post-acute care

International recommendations defined the role of physiotherapy in acute hospital care. These included respiratory management, positioning, early mobilisation and exercise prescription, with strict infection-control measures (36). The Stanford Hall consensus addressed rehabilitation after discharge, including graded return to activity (37). A rapid living systematic review by Cochrane Rehabilitation documented the growing evidence base on rehabilitation needs in patients with COVID-19 (9). WHO's pulse survey reported widespread disruption of essential health services, including rehabilitation, during 2020 (38).

**Clinical observation (author).** During the first lockdown, the author delivered home-based physiotherapy under the exceptional arrangements authorised by French health authorities. Programmes had to be adapted to the home environment with minimal equipment. Families had to be involved while infection risk was managed, and psychosocial support became part of the therapeutic encounter. These observations illustrate the practical adaptations required; they are not evaluated outcomes.

### 5.2 Post-COVID-19 condition: post-COVID functional decoupling

WHO defines post-COVID-19 condition as symptoms that usually occur three months after probable or confirmed infection, last at least two months and cannot be explained by an alternative diagnosis (39). Fatigue, post-exertional symptom exacerbation, cognitive dysfunction and dysautonomia are frequent (40). After critical illness, persistent physical, cognitive and mental impairments had already been described as post-intensive care syndrome (PICS) (41).

Within this framework, the term Post-COVID Functional Decoupling (PCFD) is proposed for a specific observation relevant to rehabilitation: the dissociation between the normalisation of physiological markers (oxygenation, imaging, inflammatory markers) and the recovery of functional capacity, which may lag behind by weeks or months. PCFD is not proposed as a new diagnosis. It is a descriptive pattern within post-COVID-19 condition and PICS, with a practical implication: functional measures, rather than physiological normalisation, should guide rehabilitation decisions and discharge. Because post-exertional symptom exacerbation may occur, exercise progression must be individualised (37, 40).

## 6 Telerehabilitation

### 6.1 The shift to remote care

Lockdowns led to the suspension of much outpatient rehabilitation and to a rapid expansion of telehealth across specialties (42). Physiotherapy was no exception, although the hands-on nature of the profession raised specific questions about the suitability of remote delivery (43).

### 6.2 Evidence of effectiveness and uptake

Table 3 summarises the evidence.

- A rapid overview concluded that telerehabilitation in physical therapy is generally comparable to in-person care for several conditions (7).
- A Cochrane review of 15 studies (1,904 participants) found that pulmonary telerehabilitation achieved outcomes similar to centre-based rehabilitation, with no safety issues, although the certainty of evidence was limited (8).
- During the pandemic, a telerehabilitation programme maintained functional capacity in patients with chronic lung disease, compared with no activity (44).
- Telerehabilitation was non-inferior to in-person rehabilitation 12 weeks after total knee arthroplasty (45).
- A mixed in-person and telerehabilitation programme was feasible in patients with cancer (46).

**Table 3:** Telerehabilitation during COVID-19: effectiveness and uptake (verified sources).

Domain	Design	Population	Key finding
Physical therapy, several conditions (7)	Rapid overview of systematic reviews	Mixed	Telerehabilitation generally comparable to in-person care
Pulmonary rehabilitation (8)	Cochrane systematic review	15 studies, 1,904 participants (mostly COPD)	Outcomes similar to centre-based rehabilitation; no safety issues; limited certainty
Chronic lung disease (44)	Quasi-experimental, retrospective (Apr 2020–Aug 2021)	32 patients	Functional capacity maintained vs. no-activity control
Total knee arthroplasty (45)	Retrospective comparative (2020)	164 patients	Non-inferior to in-person rehabilitation at 12 weeks
Outpatient physiotherapy, national database (47)	Descriptive, retrospective (2020)	222,680 patients	Telerehabilitation in 6% of episodes (10% → 5% between Q2 and Q3 2020); less used for neurological conditions
Cancer rehabilitation (46)	Prospective observational (2021–2023)	23 patients	Mixed in-person/telerehabilitation feasible

COPD, chronic obstructive pulmonary disease.

Uptake, however, was more modest than often assumed. In a US database of 222,680 outpatient physiotherapy episodes, telerehabilitation was used in 6% of cases overall, falling from 10% to 5% between the second and third quarters of 2020. It was used less for neurological conditions (47).

The term Telerehabilitation Emergency Pivot (TEP) is proposed for the system-level transition from in-person to remote rehabilitation under crisis conditions. It is characterised by speed, improvisation and the subsequent persistence of hybrid models. TEP draws on the broader literature on the digital transition of care (42). It emphasises features specific to rehabilitation, namely hands-on assessment, exercise supervision and safety, and uptake that was uneven across conditions (43, 47).

## 7 The delta-variant period (2021)

Compared with the Alpha variant, the Delta variant was associated with an approximately two-fold higher risk of hospital admission (48). It predominated during the second half of 2021, when hesitancy persisted among some health workers and the French vaccination mandate was introduced (27, 32). Long COVID appeared more frequent after Delta than after the subsequent Omicron variant (49), suggesting a sustained demand for rehabilitation after 2021.

**Clinical observation (author).** During the Delta period, the author observed younger patients with prolonged functional impairment, prominent cognitive complaints and symptoms suggesting dysautonomia among those referred for rehabilitation. These impressions come from a limited caseload and have not been quantified; they are reported as hypotheses for future studies.

## 8 Proposed constructs: positioning and testability

The four constructs are summarised in Table 4 and Figure 3. For each, the table gives a definition, the closest existing concepts, the specific analytical contribution claimed, and a possible empirical test.

**Table 4:** Proposed constructs: definition, positioning, claimed contribution and testability.

Construct	Working definition	Closest existing concepts	Claimed contribution	Possible empirical test
Frontline Abandonment Syndrome (FAS)	Withdrawal (temporary or prolonged, formal or informal) of health workers from high-risk front-line duties, with consequences for continuity of care	Burnout (20); moral injury (21, 22); compassion fatigue (23); duty-to-care ethics (24)	Shifts attention from the individual psychological state to the observable organisational consequence (withdrawal) and its determinants (PPE, communication)	Linkage of staffing, absence and redeployment data with PPE availability and validated burnout or moral-injury scores during surges
Vaccination Refusal Cascade (VRC)	Hypothesised sequence in which a salient institutional or media event triggers successive refusal decisions within professional networks	Vaccine hesitancy (25); opinion clustering (34); social influence (35)	Specifies a trigger event and diffusion within professional networks, rather than generic hesitancy	Interrupted time-series of vaccination intention or uptake among health workers around safety alerts; network analyses within institutions
Telerehabilitation Emergency Pivot (TEP)	System-level transition from in-person to remote rehabilitation under crisis conditions, followed by partial persistence of hybrid models	Telehealth and digital transition of care (42); telerehabilitation effectiveness (7, 8)	Highlights constraints specific to rehabilitation (hands-on assessment, exercise supervision, safety) and uptake that varies by condition (43, 47)	Adoption curves by condition and setting; comparison of outcomes and safety between in-person, remote and hybrid episodes
Post-COVID Functional Decoupling (PCFD)	Dissociation in which physiological markers normalise while functional capacity recovers weeks to months later	Post-COVID-19 condition (39); long COVID (40); post-intensive care syndrome (41)	A descriptive, rehabilitation-oriented pattern within existing definitions; supports function-based rehabilitation and discharge criteria	Cohort studies comparing time to physiological normalisation with time to functional recovery (e.g., 6MWT, ADL scales)

All four constructs are heuristic proposals derived inductively; none has undergone formal concept analysis (13) or empirical validation.

**Figure 3:**
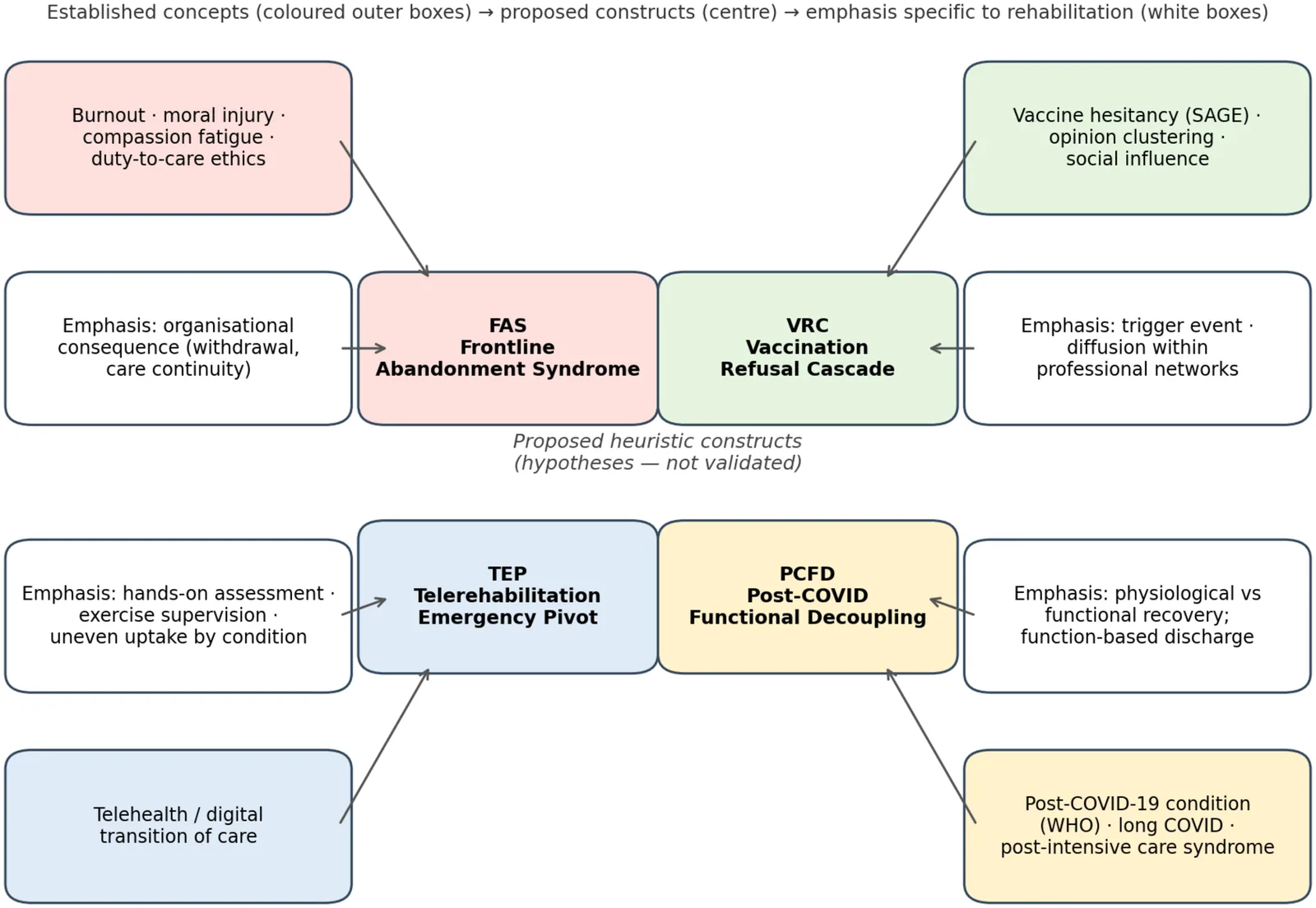
Conceptual map positioning the four proposed constructs (centre) relative to the closest established concepts (outer boxes) and to the rehabilitation-specific elements they emphasise. The references for each established concept are given in Table 4.

These constructs are presented as heuristic proposals and hypotheses. They are not validated constructs, and none replaces existing terms. Their claimed contribution is to shift attention to phenomena that are meaningful for rehabilitation services:

- from the individual psychological state (burnout, moral injury) to the organisational consequence of withdrawal (FAS);
- from general social contagion to trigger-specific diffusion within professional networks (VRC);
- from the digital transition in general to the constraints specific to rehabilitation (TEP);
- from symptom-based definitions of post-COVID-19 condition to the dissociation between physiological and functional recovery (PCFD).

Formal concept analysis (13) and empirical studies are needed before these terms can be recommended for wider use.

## 9 Discussion

This review brings together four dimensions of the pandemic that are usually studied separately, from the point of view of rehabilitation. Three lessons stand out for pandemic preparedness.

(1) **Health-worker protection and monitoring.** The gap between reported and estimated deaths (14) and the increased risk associated with inadequate protective equipment (16) support systematic monitoring of occupational infections and deaths. They also support the inclusion of rehabilitation staff in protection plans.
(2) **Communication about vaccination.** The occupational gradient and the sensitivity of vaccination intention to media controversies (26, 27) suggest that communication strategies should target health-worker groups with lower acceptance, before and during safety alerts.
(3) **Rehabilitation in preparedness plans.** Rehabilitation needs extend well beyond the acute phase (37, 39). Telerehabilitation can maintain continuity for suitable patients (7, 8), but its limited uptake for some conditions (47) argues for hybrid models rather than wholesale substitution.

For Africa, where fewer than one in ten health workers had been fully vaccinated by September 2021 (15), these lessons are particularly relevant. Rehabilitation data from sub-Saharan Africa during the pandemic remain scarce.

## 10. Limitations

The main limitations are the following:

- **Design.** This is a narrative review with purposive selection. It is not exhaustive, is exposed to selection and publication bias, and includes no formal quality appraisal of the individual studies.
- **Comparability of data.** Hesitancy estimates use different definitions and periods and cannot be compared directly.
- **Clinical observations.** They reflect a single observer in a single high-resource setting and are subject to recall bias. They are separated from the evidence but could still influence how it is interpreted.
- **Proposed constructs.** They were derived inductively without formal concept analysis and have not been tested empirically.
- **African data.** Rehabilitation data from sub-Saharan Africa are lacking.
- **Period covered.** The review focuses on 2020–2021, whereas the management of post-COVID-19 condition and the integration of telerehabilitation continue to evolve.

## 11. Conclusion

COVID-19 revealed the vulnerability of health workers, the fragility of confidence in vaccination within professional communities, and the essential role of rehabilitation across the acute, post-acute and community phases. Telerehabilitation proved a useful complement to in-person care, but not a universal substitute. The four constructs proposed here are hypotheses intended to stimulate formal concept analysis and empirical research, particularly on workforce retention, communication about vaccination, hybrid rehabilitation models and functional recovery after COVID-19.
